# Digestibility and Nutritional Value of Microalga *Tetraselmis* sp. for Weaner Piglets

**DOI:** 10.3390/ani15070967

**Published:** 2025-03-27

**Authors:** Andreia A. M. Chaves, Cátia F. Martins, David M. Ribeiro, Margarida R. G. Maia, António J. M. Fonseca, Ana R. J. Cabrita, Susana P. Alves, Miguel P. Mourato, Mário Pinho, Rui J. B. Bessa, André M. de Almeida, João P. B. Freire

**Affiliations:** 1 LEAF—Linking Landscape, Environment, Agriculture and Food Research Center, Instituto Superior de Agronomia, Universidade de Lisboa, Tapada da Ajuda, 1349-017 Lisboa, Portugal; andreiachaves@isa.ulisboa.pt (A.A.M.C.); catiamartins@isa.ulisboa.pt (C.F.M.); davidribeiro@isa.ulisboa.pt (D.M.R.); mmaia@isa.ulisboa.pt (M.R.G.M.); mmourato@isa.ulisboa.pt (M.P.M.); jpfreire@isa.ulisboa.pt (J.P.B.F.); 2 Associate Laboratory TERRA, Instituto Superior de Agronomia, Universidade de Lisboa, Tapada da Ajuda, 1349-017 Lisboa, Portugal; 3 REQUIMTE, LAQV, ICBAS, School of Medicine and Biomedical Sciences, University of Porto, Rua Jorge Viterbo Ferreira, 228, 4050-313 Porto, Portugal; ajfonseca@icbas.up.pt (A.J.M.F.); arcabrita@icbas.up.pt (A.R.J.C.); 4 CIISA—Centro de Investigação Interdisciplinar em Sanidade Animal, Faculdade de Medicina Veterinária, Universidade de Lisboa, Avenida da Universidade Técnica, 1300-477 Lisboa, Portugal; susanaalves@fmv.ulisboa.pt (S.P.A.); mpinho@fmv.ulisboa.pt (M.P.); 5 AL4AnimalS—Associate Laboratory for Animal and Veterinary Sciences, Avenida da Universidade Técnica, 1300-477 Lisboa, Portugal

**Keywords:** digestible energy, fatty acids, feed evaluation, microalgae, minerals, post-weaning piglets, total tract apparent digestibility

## Abstract

Twenty-four male piglets were randomly assigned to four dietary treatments, with increasing *Tetraselmis* sp. incorporation levels (0%, 5%, 10%, and 15%). Following a 4-day adaptation period to metabolic cages, the animals were used in a 2-week digestibility trial and slaughtered for digestive tract measurements and sampling. The apparent total tract digestibility (ATTD), N balance, small intestine digesta viscosity and histomorphology, and hindgut digesta volatile fatty acid (VFA) profile were determined. Polynomial contrasts were employed to examine linear and quadratic effects of *Tetraselmis* sp. dietary incorporation. The ATTD of most macronutrients and N retention efficiencies decreased linearly (*p* < 0.05) with *Tetraselmis* sp. dietary inclusion. The ileum villi height increased linearly (*p* < 0.001) and the hindgut VFA concentration increased linearly (*p* < 0.05) with dietary *Tetraselmis* sp. inclusion. The ATTD values estimated for *Tetraselmis* sp. biomass using the regression method were 68.3% ± 3.86 for dry matter (DM), 66.1% ± 5.11 for N, and 61.3% ± 4.28 for gross energy. The values calculated for digestible and metabolizable energy (MJ/kg DM) and digestible crude protein (% DM) for *Tetraselmis* sp. were 9.0, 8.8, and 18.3, respectively. *Tetraselmis* sp. biomass had lower ATTD values when compared to protein sources commonly used in swine nutrition.

## Simple Summary

One of the most significant challenges in present-day animal nutrition is the establishment of novel sustainable ingredients, particularly protein sources. Due to their promising composition and local production, microalgae are considered a suitable alternative feedstuff. However, a significant gap in the available information about the feeding value of microalgae still remains, with no thorough characterization of their nutritional value in the framework of swine nutrition. This information is nonetheless essential for its practical use in diet formulations for this species. This research addresses this gap by evaluating the nutritional value of *Tetraselmis* sp. biomass when included in the diets of weaner piglets up to 15%, thus ascertaining its relevance to the nutrition of these animals.

## 1. Introduction

Meat production, particularly poultry and pork, is expected to continually increase to meet the growing demands driven by demography and improvements in living standards, particularly in developing countries [1]. Feedstuff availability, particularly protein sources, must also increase in order to support the growing demand for meat products, competing with other uses, particularly human nutrition [2]. This scenario renders necessary the establishment of alternative protein sources. Microalgae, due to their usually high protein content, have received growing interest as a potential alternative feedstuff for monogastric nutrition, and their incorporation in swine and poultry diets has been studied in recent years [3,4].

In general, microalgae are considered valuable feed sources due to their high protein, vitamins, minerals, and essential fatty acid contents [5,6]. Also, the existence of various and valuable bioactive compounds in these microalgae, with antioxidant, anticarcinogenic, and antimicrobial effects, increases their value for all industries, including animal nutrition [7,8]. However, their nutritional composition can vary widely depending on species, strain, and production conditions under which they are cultivated or produced [9]. Many species also exhibit antioxidant and anti-inflammatory properties that can enhance animal health and welfare [10]. Moreover, microalgae cultivation presents environmental advantages as it can be locally produced, requiring minimal land and freshwater uses, thus alleviating pressure on natural resources [11,12]. Nonetheless, the production processes face numerous challenges, particularly the high costs involved in the drying process and composition variability [3].

Microalgae can be included in the diet of weaned piglets as a sustainable and nutritionally rich ingredient or supplement with potential health-promoting proprieties [3]. However, microalgae feeding might also lead to digestive disorders due to mineral imbalance and anti-nutritional or toxic compounds [13].

*Tetraselmis* sp. has recently attracted attention for industrial use, particularly due to its rapid growth rate and high protein, long-chain n-3 polyunsaturated fatty acids (n-3 PUFA), and vitamin E content with potential antimicrobial and probiotic properties [14]. However, and according to the latter authors, *Tetraselmis* sp. biomass has a high mineral content that may decrease its organic matter and nutritional value. The unique cell wall of *Tetraselmis* sp., or theca, composed of fused scales [15], may also limit its nutritional value by decreasing access to intracellular nutrients [16].

Despite the potential advantages and limitations of *Tetraselmis* sp. for animal nutrition, and as for microalgae in general, there is a lack of information in the currently available literature regarding its nutritional value for piglets, including its digestibility and energy values. We recently evaluated the nutritional value of the microalga *Nannochloropsis oceanica* for weaner pigs [17], and in this research, we report the nutritional evaluation of the *Tetraselmis* sp. for weaner piglets.

Herein, we determine the nutritional value of spray-dried *Tetraselmis* sp. biomass by assessing its digestible energy (DE), metabolizable energy (ME), and digestible crude protein (DCP) for weaner piglets. Furthermore, this research explores the suitability of microalgae as a feedstuff for piglet nutrition during the critical post-weaning stage.

## 2. Materials and Methods

### 2.1. Animals and Diets

All the procedures were revised by the Ethics Commission of Instituto Superior de Agronomia (ISA) and approved by the Portuguese National Veterinary Authority’s Animal Care Committee (process number 0421/000/000/2019), following national and European Union legislation (2010/63/EU Directive). The experiment took place at the ISA experimental facilities of the University of Lisbon (Lisbon, Portugal) in February 2020.

Twenty-four male piglets [Pietrain × (Large White × Landrace)] were acquired from a commercial farm and transferred to the ISA facilities two weeks after weaning (weaning at 28 days of age). At the experimental facilities, the animals were weighed and housed individually in metabolic cages (1000 × 500 × 480 mm) with an infrared heating lamp and free access to water, as described by Chaves et al. [17]. The experiment started after 4 days of adaptation. The piglets had an initial average body weight of 11.7 kg ± 1.34 (mean ± SD) and were randomly assigned to one of four dietary treatments (*n* = 6): Control (a diet based on wheat, corn, and soybean meal), TSM5 (95% control diet and 5% *Tetraselmis* sp.), TSM10 (90% control diet and 10% *Tetraselmis* sp.), and TSM15 (85% control diet and 15% *Tetraselmis* sp.). The microalga was added progressively by replacing the basal diet to assess its nutritional contribution through regression analysis. The *Tetraselmis* sp. microalga biomass used in this experiment was produced in closed tubular photobioreactors and supplied as a feed-grade spray-dried powder by Allmicroalgae—Natural Products SA (Pataias, Portugal); it was mixed into the diets, which were then pelleted. Table 1 and Table 2 show the composition of the diets and the microalga, respectively. The experiment lasted 14 days.

### 2.2. Feed Intake, Growth, Urine, and Feces Sample Collection

Throughout the experiment, feed intake was controlled and equalized between groups using a restriction approach. During the first week of the experiment, each group was fed with 3.9 kg (as-fed basis) of the diet. In the second week, this amount was increased to 4.2 kg (as-fed basis). To assess the apparent total tract digestibility (ATTD) and the N balance of the diets throughout the experiment, feed intake, refusals, total feces, and urine were collected, weighed, and recorded daily for each animal. A sample from each diet and refusal and a composite sample of feces and urine were retrieved. Briefly, the total feces produced were collected daily, individually for each animal, weighed, and stored. At the end of each trial week, a composite fecal sample was made and stored for further analysis (at −20 °C). The same was conducted for urine. To prevent N loss, in the urine samples, 50 mL of 5% sulfuric acid was added to the collection container daily. The animals were weighed on days 0, 7, and 14 of the experiment before feeding. Daily dry matter (DM) intake and average daily gain (ADG) were calculated as described by Chaves et al. [17].

During the experiment, four animals, one from each treatment, died from unknown causes: one from the Control group at the end of the 1st week, and one from each other three groups (TSM5, TSM10, and TSM15) at the end of the 2nd week. Therefore, 20 animals remained in this study, 5 per group (*n* = 5), with the groups thus remaining balanced.

### 2.3. Slaughtering and Sampling

At the end of the experiment, animals were slaughtered via exsanguination following prior electrical stunning and were subsequently eviscerated following appropriate legislation. The contents of the stomach, duodenum plus jejunum, ileum, caecum, and colon were collected, and their viscosity and pH determined. Additionally, the caecum and colon contents samples were preserved in 5% (*v*/*v*) ortho-phosphoric acid at −20 °C for volatile fatty acid (VFA) determination. The gastrointestinal tract was weighed and measured. The duodenum (10 cm below pylorus), jejunum (5.5 m below pylorus), and ileum (60 cm above ileum–caeca valve) were the small intestine segments obtained for histological analysis. Immediately after collection, tissues were submerged in a 10% formalin solution, and after that, they were prepared for paraffin embedding.

### 2.4. Microalga, Diet, Feces, and Refusal Analyses

The fecal samples were dried at 60 °C for 72 h, and refusals at 65 °C for 48 h, in a ventilated oven and then finely ground (1 mm mesh). Analyses were conducted on the microalga, diet, and fecal samples following standard procedures [19]: DM (method 935.29), ash (method 942.05), ether extract (EE, method 920.39), and Kjeldahl N (method 954.01) contents. Crude protein (CP) was calculated as N × 6.25 for diets and feces [19] and as N × 4.78 for microalgae [18]. The neutral detergent fiber (NDF; with heat-stable amylase and without sodium sulfite) and acid detergent fiber (ADF) contents of the feces, diets, and microalgae were obtained sequentially according to Van Soest et al. [20] and expressed inclusive of residual ash. The lignin content of the diets was also determined [21]. The filtration step utilized in NDF and ADF content determination was achieved through a glass microfiber filter due to the small particle size of the microalgae, as described by Cabrita et al. [22]. The gross energy (GE) content was determined by complete combustion in a Parr 1261 adiabatic bomb (Parr Instrument Company, Moline, IL, USA). For the refusals, only DM was performed as previously described.

The fatty acid methyl esters (FAMEs) from the microalga, diets, and feces were obtained through direct transesterification with sodium methoxide in methanol followed by hydrochloric acid in methanol and analyzed using gas chromatography with flame ionization detection (GC-FID) equipped with a capillary column (SP-2560, 100 m × 0.25 mm × 0.20 μm; Supelco Inc., Bellefonte, PA, USA), following the method described by Alves et al. [23]. An internal standard (methyl nonadecanoate, Sigma-Aldrich, St. Louis, MO, USA) was added to the samples before transesterification. The mineral profile from the microalga, diets, and feces was determined using inductively coupled plasma–optical emission spectrometry according to the method described by Ribeiro et al. [24]. This analysis was carried out on an iCAP 7200 duo, ICP-OES spectrometer (Thermo Scientific, Waltham, MA, USA) equipped with an automated sampler.

The fecal samples were analyzed in duplicate, while the diet and microalga samples were analyzed in triplicate.

### 2.5. Urine Sample Analysis

After thawing the composite urine samples, the N content was obtained using the Kjeldahl method [19]. Then, the urine samples were freeze-dried (CoolSafe Superior Touch 95 freeze-dryer, Labogene Alleroed, Lillerød, Denmark) at −92 °C and 0.2 hPa for 5 days, and the GE content was analyzed. Both analyses were performed in duplicate per sample.

### 2.6. Intestinal Content Analysis

Viscosity determinations were carried out on an LVDVCP-II viscometer (Brookfield Engineering Laboratories, Middleboro, MA, USA) adjusted to 6 rpm and 23 °C, as described by Martins et al. [25]. In summary, the samples of the small intestine contents were centrifuged at 18,144× *g* for 10 min in a J2-HS ultracentrifuge (Beckman-Coulter, Brea, CA, USA), and the supernatant viscosity was measured in duplicate.

The pH values of the stomach, duodenum with jejunum, ileum, caecum, and colon contents were measured using a glass electrode pH meter (Metrohm 744, Metrohm AG, Herisau, Switzerland) immediately after collection.

For VFA determinations, the samples were thawed and analyzed using a Shimadzu 2010Plus chromatograph (Shimadzu Corp., Kyoto, Japan) equipped with a flame ionization detector and a fused capillary column (Nukol, 30 m length × 0.25 mm internal diameter × 0.25 μm film thickness, Supelco Inc.) [26]. The VFAs were quantified based on an internal standard (4-methylvaleric acid, Sigma-Aldrich, St. Louis, MO, USA).

### 2.7. Intestinal Histomorphologic Analysis

Tissue samples, cut to a thickness of 7 μm, were examined under a microscope after pre-staining with hematoxylin–eosin to assess villi heights and widths, as well as crypt depths. The examination was conducted using a BX 51 microscope (Olympus, Tokyo, Japan) equipped with 4× and 10× lenses. Subsequently, digital images were captured using a DP 21 camera (Olympus). Olympus DP-Soft software (Olympus) was used to determine the height and width of the villi and the depth of the crypts.

### 2.8. Calculations and Statistical Analysis

The ATTD was calculated by dividing the difference between the nutrients ingested and excreted in feces over the nutrients ingested. The DE was calculated by subtracting from the GE intake the GE excreted in the feces. The ME was obtained by subtracting from the GE intake the GE excreted in the feces and urine. The data were assessed for normality and homogeneity of residual variances. The Proc MIXED of the SAS software package (version 9.4; SAS Institute Inc., Cary, NC, USA) was used to analyze the data. The model for the ATTD, fecal DM, and N balance data included the week of the sampling period (Wk), diet (D), and their interaction as fixed effects, considering the repeated measurements in the week for each piglet. For the remaining variables, a simple model considering only dietary treatment as a fixed effect was used. Polynomial contrasts were applied to test the linear and quadratic effects of increasing levels of the microalga in the diet. Statistical significance was declared at *p* < 0.05.

When a linear effect of dietary *Tetraselmis* sp. incorporation on the ATTD of nutrients was detected, a mixed model was fitted, where the proportion of *Tetraselmis* sp. in the diet was treated as a continuous variable, the week of measurements was a fixed effect, and the animal was included as a random factor. The ATTD of *Tetraselmis* sp. biomass was estimated, and its confidence limits were obtained by solving the regression model for 100% *Tetraselmis* sp. incorporation using the “estimate” function in Proc MIXED.

## 3. Results

### 3.1. Feed Intake, Live Weight, and Fecal Dry Matter

Table 3 presents the effect of increasing *Tetraselmis* sp. dietary inclusion on feed intake, live weight, and fecal DM. The DM intake and ADG decreased linearly (*p* < 0.05), while the final live weight (LW) tended to decrease (*p* = 0.087) with *Tetraselmis* sp. dietary incorporation, with a reduction in DM intake, ADG, and LW between the Control group and the TSM15 group of 114 g/d, 131 g/d, and 2.5 kg, respectively.

The fecal DM did not differ among the treatments. However, the fecal DM was lower in the first week than in the second week (294 vs. 323 g/kg, *p* = 0.027), and no significant interactions between diet and period were observed.

### 3.2. ATTD, N Retention, and Energy Values of the Diets

Table 4 and Table 5 show the effects of incorporating *Tetraselmis* sp. on the ATTD, DE, and ME of the diets and the N balance of the animals in this study. The dietary incorporation of *Tetraselmis* resulted in a linear decrease (*p* < 0.01) in the ATTD of DM, OM, N, and GE and a quadratic decrease in EE (*p* = 0.022). The dietary incorporation of *Tetraselmis* sp. increased linearly the ATTD of ashes (*p* < 0.001) and quadratically the NDF (*p* = 0.014), whereas the ADF remained unaffected. The decrease in the ATTD between Control and TSM15 was circa 3 percentual points for DM and OM, 4 percentual points for GE, and 6 percentual points for EE.

The ATTD of all the FAs decreased linearly with the incorporation of *Tetraselmis* sp. in the diet (*p* < 0.001), except for 20:5n-3, where no differences were observed (*p* = 0.696; Table 4).

The ATTD of the reported minerals was unaffected by the diets, except for sulfur, which showed a significant linear decrease in response to the increased *Tetraselmis* sp. incorporation (*p* = 0.005; Table 4).

Nitrogen intake tended (*p* = 0.058) to decrease with the *Tetraselmis* sp. dietary inclusion level, and N retained, the N retention coefficient (NRC), and the practical N retention coefficient (NPRC) decreased linearly (*p* < 0.05; Table 5). The urinary excreted N increased linearly (*p* = 0.001) with *Tetraselmis* sp. dietary inclusion. NRC is the proportion of absorbed N that was retained, and NPRC represents the proportion of ingested N that was retained. The animals fed the TSM15 diet had a decrease of 10 percentual points of both parameters when compared to the Control animals.

The contents of DE and ME of the diets decreased linearly (*p* < 0.001) with *Tetraselmis* sp. dietary inclusion. Moreover, the ME/DE ratio also displayed the same pattern (*p* < 0.001; Table 5).

### 3.3. Estimated ATTD and Energy Content for Tetraselmis sp. Biomass

The ATTD estimates, using the regression approach, for *Tetraselmis* sp. biomass are shown in Table 6. The estimated ATTD values for DM and OM were 68.3% ± 3.86 and 68.4% ± 3.57, respectively. The ATTD of N and GE were slightly lower, at 66.1% ± 5.11 and 61.3% ± 4.28, respectively, while for EE, it was notably lower, at 41.1% ± 9.55.

The estimated ATTD values for the total FAs were quite low (26.4 ± 6.3), reflecting the wide range of ATTD values estimated for the individual FAs (from −638% for 18:0 up to 99% for 20:5n-3). Nevertheless, the ATTD estimates for the n-3 PUFA characteristics of this microalga (18:3n-3, 20:5n-3) showed high values above 90%.

In the fecal samples, we detected the presence of odd-chain FAs, branched-chain FA trans-18:1 isomers, and dimethyl esters that were not present in the diets. Notably, the trans-18:1 isomers were not negligible and summed 5.4, 7.7, 12.0, and 10.2% of the total FAs, respectively, for Control, TSM5, TSM10, and TSM15 (linear effect, *p* = 0.007).

The estimated DE content of biomass was 9.04 MJ/kg DM, while the ME was 8.84 MJ/kg DM. Finally, the DCP calculated for the *Tetraselmis* sp. biomass was 18.3% DM.

### 3.4. Viscosity and pH of Gastrointestinal Contents and Intestine Histomorphology

Table 7 shows the data on the viscosity and pH of the gastrointestinal contents, as well as the histomorphology of the small intestine tissues. No significant differences were found in viscosity contents of the two compartments measured (*p* > 0.05). As for pH, only the duodenum + jejunum content showed a linear increase (*p* = 0.002) with *Tetraselmis* sp. dietary inclusion. No significant differences were observed for the pH of the contents of the other compartments (*p* > 0.05).

The incorporation of *Tetraselmis* sp. in the diet did not affect the small intestine histomorphology variables, except for the linear increase in villi height (*p* < 0.001) and consequent increase in the villi/crypt ratio (*p* = 0.032) in the ileum.

### 3.5. Volatile Fatty Acid Profile

Table 8 shows the VFA content and profile found in the caeca and colon digesta. Regarding the cecal digesta and total VFA concentration (mmol/L), no differences (*p* > 0.05) were observed with the increased *Tetraselmis* sp. incorporation. The molar proportion of butyric acid (C4:0) and valeric acid (C5:0) decreased linearly with the *Tetraselmis* sp. incorporation (*p* < 0.05), while the proportion of iso-butyric acid (iso-C4:0) and iso-valeric acid (iso-C5:0) increased linearly with the dietary *Tetraselmis* sp. inclusion level (*p* < 0.05).

Regarding the colon digesta, the total VFA concentration (mmol/L) showed a linear increase (*p* = 0.023) with the *Tetraselmis* sp. dietary inclusion level. Concerning the molar proportions, the only effect observed was a significant linear decrease in caproic acid (C6:0) with a decrease in the *Tetraselmis* sp. inclusion level (*p* = 0.006).

## 4. Discussion

Few studies have yet evaluated algae species as alternative ingredients for weaner piglets. To the best of our knowledge, this experiment is the first to evaluate the ATTD as well as the DE and ME contents of *Tetraselmis* sp. biomass in piglet diets. A similar study was conducted by Brugger et al. [27] on the macroalga *Laminaria japonica*, and more recently, our team assessed the microalga *Nannochloropsis oceanica* [17]. Regarding specifically *Tetraselmis* sp. biomass, studies on its nutritional value are mostly limited to its use as aquafeeds [28,29,30,31]. Thus, research focusing on the nutritional characterization of different microalgae species on farm animals is still very limited, leading to a significant lack of information regarding such novel and potential feed ingredients.

In this experiment, the increased dietary incorporation of *Tetraselmis* sp. (up to 15%) allowed us to evaluate the linearity of the nutrient ATTD responses. For the variables that exhibited a linear ATTD response, we estimated the ATTD through linear extrapolation to a theoretical scenario of 100% *Tetraselmis* sp. biomass. Nevertheless, the ATTD values estimated for *Tetraselmis* sp. biomass should be interpreted within the incorporation range studied (0 to 15%). As this range includes the majority of practical applications, the estimated ATTD values are likely appropriate for use in the additive framework of feed formulation.

It is important to highlight that this experiment was a short-term digestibility trial, conducted with a restricted feeding regime, which is normal practice in this type of study in order to guarantee uniform feed intake through treatments; thus, it did not aim to evaluate piglet growth performance. However, the linear decreases in DM intake and ADG observed with the increasing inclusion levels of *Tetraselmis* sp. suggests that it may decrease the diet acceptability and its nutritional value. Nevertheless, *Tetraselmis* sp. biomass has never been used in swine production trials; thus, its effect on productive performance has not been evaluated yet.

The observed linear reductions in the ATTD of DM, OM, N, and GE of the diets with increasing *Tetraselmis* sp. inclusion support findings from previous studies by our team that noted decreased digestibility values when other microalgae (*Chlorella vulgaris* and *Spirulina platensis*) were added to piglets’ diets [25,32].

The ATTD of DM, OM, N, and GE estimated for the *Tetraselmis* sp. biomass are all relatively low (ranging between 61% for GE and 68% for OM). This contrasts with the ATTD values above 92% for OM, GE, and CP observed for skimmed milk powder commonly used in piglet starter diets [33]. Common plant-based protein sources, like soybean meal, also show higher ATTD values, approximately 86% in growing pigs for OM, GE, and N [33]. Even when compared to other green eukaryotic microalgae, such as *Nannochloropsis oceanica*, which showed values around 73%, 71%, and 83%, respectively, for DM, OM, and N ATTD [17], *Tetraselmis* sp. has lower digestibility.

The observed lack of an effect for ADF ATTD and the quadratic increase effect for NDF ATTD is difficult to explain and possibly can be attributed to methodological issues due to either the low fiber content of the weaner’s diets or particularly to difficulties in fiber determinations for seaweeds and microalgae [17,34]. This challenge arises because traditional methods, such as those by Van Soest, with NDF and ADF determinations, were developed for terrestrial plants and are not fully effective for determining the complex and diverse polysaccharide content of microalgae cell walls. Moreover, in some cases, microalgae contain mineral components that interfere with the accuracy of such methods [35]. Thus, the application of conventional methods to microalgae often results in errors and inconsistencies, underestimating or omitting important fiber fractions. Therefore, to obtain precise and reproducible results, it is necessary to develop analytical methods that take into account the unique structural characteristics of microalgae.

The increased dietary *Tetraselmis* sp. inclusion decreased linearly the N intake, N retained, and N retention efficiencies. The reduction in N retention efficiencies and the increase in urinary N excretion indicates that incorporating *Tetraselmis* sp. decreases the apparent protein biological value of the diets probably due to an unbalanced amino acid profile [36,37]. The analysis of the amino acid content and profile of *Tetraselmis* sp. and the amino acid ileal digestibility should be addressed in future experiments to clarify this point. In addition, post-harvest treatments, such as physical-mechanical disruption or enzymatic methods, have been reported to increase *Tetraselmis* sp. ATTD in feeds for European seabass juveniles [28].

The DE and ME contents of the diet also decreased linearly with increasing levels of *Tetraselmis* sp., which is consistent with the lower GE ATTD values observed. The reduced ME/DE ratio is consistent with the decrease in the apparent protein biological value (i.e., NRC) and associated increased urinary energy losses. The estimates of DE (9 MJ/kg DM) and ME (8.8 MJ/kg DM) obtained for the *Tetraselmis* sp. biomass are quite low, even when compared to those values obtained by us for the microalgae *N. oceanica* (12.7 for DE and 12.4 for ME) [17]. Such low DE and ME contents are explained by the high ash content (33.4% DM) and the low ATTD of EE and total FAs. The mineral content explains only approximately 33% of the ash content of *Tetraselmis* sp. biomass. Such discrepancy between ash and total mineral contents in microalgae has been reported by other authors [17,22,38]. Furthermore, the latter authors [38] suggested that this could be due to the presence of sandy particulates and silica. Moreover, it is not clear why, despite the increase in ash ATTD with increased dietary *Tetraselmis* sp. biomass incorporation, the digestibility of almost all individual minerals determined did not show significant differences between diets. This apparent inconsistency in the mineral content and ATTD warrants further studies with refined methodologies in order to be fully understood. Notably, sulfur was the only mineral that decreased the ATTD with the increased dietary *Tetraselmis* sp. incorporation level. Sulfur is an important component of proteins; thus, the decrease in its ATTD could be directly related to the decrease in protein ATTD. Sulfur is a crucial element in certain amino acids and vitamins, and its reduced bioavailability and absorption may negatively impact protein metabolism and N retention [39,40].

We attempted to estimate the ATTD of the individual FAs of *Tetraselmis* sp. biomass, but the results vary widely. Stearic acid (18:0) presented a consistently negative ATTD, which is in accordance with the presence of trans-18:1 isomers in feces (ranging from 5.4 up to 12.0% of the total FAs). The presence of trans-18:1 in the digestive tract of animals is indicative of the occurrence of biohydrogenation of dietary unsaturated C18 FA that yields, as an end product, 18:0. The production of 18:0 within the digestive tract of weaners results in the observed negative ATTD of 18:0. We also report the presence of high trans-18:1 isomers and a very low ATTD of 18:0 (estimates ranging from 3.5 to 20%) for *N. oceanica* [17]. In this experiment, the 18:0 ATTD is overtly negative, and this difference might be explained by the low proportion of C18 unsaturated FAs (substrates for the biohydrogenation) of *N. oceanica* (≈8% of the total FAs) [17] rather than of the *Tetraselmis* sp. biomass (≈50% of the total FAs). Nevertheless, the ATTD obtained for 16:0 is also negative, and this could not be explained easily through the biohydrogenation of unsaturated C16 FAs as they are present in low concentrations. The estimates for the 18:3n-3 and 20:5n-3 ATTD of the *Tetraselmis* sp. biomass are quite high; however, due to the apparently extensive biohydrogenation, this does not necessarily represent the net absorption of these PUFAs. However, the inclusion of *Tetraselmis* sp. biomass in the diets may be expected to improve meat quality and animal health, considering its high content of omega-3 PUFAs [7].

During piglet weaning, with drastic changes from milk to a solid diet, a depressing effect on the digestive capacity of the small intestine with a decrease in villi height and an increase in crypt depth is commonly reported [41]. The inclusion of *Tetraselmis* sp. did not alter the intestine histomorphological traits except for the beneficial effect on the increased villi heights in the ileum and consequent increase in the villi/crypt ratio. Thus, this indicates that the poor nutritional evaluation of the *Tetraselmis* sp. biomass is due mostly to microalgae intrinsic factors linked to its composition, notably, the high ash content and low digestibility of its nitrogenous compounds, rather than to a deleterious effect on the intestinal mucosa.

Moreover, the high dietary mineral content, in particular, sodium, phosphorus, and potassium, could contribute to the elevated pH of the duodenum and jejunum digesta [42,43]. As the small intestine is important in mineral absorption, the high dietary mineral content might lead to an increase in villi height in the ileum in order to couple with increased mineral availability [44].

The VFA concentration in the large intestine can be difficult to interpret as determinant factors like VFA production, absorption, and flow are not known. Thus, assuming constant VFA absorption, the increased VFA concentration observed in the colon digesta of animals fed increasing amounts of *Tetraselmis* sp. biomass might be a consequence of either increased production and/or decreased digesta flow as a consequence of reduced DM intake [45]. The cecum is an anatomical structure designed to ensure delay of digesta transit; thus, it would be less affected by the DM intake in the digestive tract flow, and this might explain the absence of an effect on the VFA concentration. Nevertheless, although there was no change in the VFA concentration in the cecum, a change in the VFA profile was also observable, with a decrease in C4:0 and C5:0 and increased branched-chain VFAs (iso-C4:0 and iso-C5:0). The branched-chain VFAs derive from the fermentation of the branched-chain amino acids valine and leucine and, hence, of protein fermentation [46,47] The increase in the branched-chain VFAs probably reflects the increased availability of these amino acids as a consequence of presumably decreased protein digestibility in the small intestine.

Besides the incomplete nutritional information addressed in the present experiment, the practical utilization of *Tetraselmis* sp. continues to be difficult due to the variability in production batches and the high cost of production [17,48]. This is a problem common to most microalgae. It is thus crucial to obtain better nutritional characterizations of the potentially interesting microalgae, improve the homogeneity in the biomass batches, and reduce production costs in order to be competitive with other conventional feedstuffs.

## 5. Conclusions

Incorporating up to 15% of *Tetraselmis* sp. biomass into weaned piglet diets resulted in a consistent reduction in the ATTD of most macronutrients. The nutrient ATTD of *Tetraselmis* sp. was calculated using a regression approach, allowing for the determination of its DM (68.3% ± 3.9) and CP (66.1% ± 5.1) digestibility and its DE (9.0 MJ/kg DM ± 0.64), ME (8.8 MJ/kg DM ± 0.62) and DCP (18.3% DM) content. These nutritional evaluation parameters are of paramount importance to allow the use of any feedstuff by the feed industry regarding diet formulation. Given the developing digestive system of piglets, these findings support the dietary inclusion of *Tetraselmis* sp. at low levels.

## Figures and Tables

**Table 1:** Ingredient composition, analytical chemical composition, gross energy content, fatty acid profile, and mineral content of the diets.

	Diets ^1^			
	Control	TSM5	TSM10	TSM15
**Ingredient (% as fed basis)**				
Wheat	43.7	41.5	39.0	37.0
Corn	15.0	14.2	13.5	12.7
Soybean meal	25.0	23.7	22.5	21.0
Dry milk	10.0	9.5	9.0	8.5
Vegetable oil	3.0	2.8	2.7	2.5
*Tetraselmis* sp.	0.0	5.0	10.0	15.0
L-Lysine ^2^	0.5	0.5	0.5	0.5
DL-Methionine ^3^	0.1	0.1	0.1	0.1
Threonine ^4^	0.1	0.1	0.1	0.1
Calcium carbonate	0.5	0.5	0.5	0.5
Dicalcium phosphate	1.3	1.3	1.3	1.3
Sodium chloride	0.3	0.3	0.3	0.3
Mineral and vitamin complex ^5^	0.5	0.5	0.5	0.5
**Chemical composition (% DM)**				
Dry matter (%)	89.6	89.6	89.5	89.8
Ash	5.8	7.3	8.3	10.3
Crude protein (N × 6.25)	20.8	21.8	22.6	23.0
Crude protein adjusted ^6^	20.8	21.1	21.5	21.8
Ether extract	5.4	5.5	5.4	5.9
Neutral detergent fiber	12.0	12.1	12.7	11.8
Acid detergent fiber	3.7	3.3	2.9	3.0
Lignin	0.65	0.69	0.86	0.74
Gross energy (MJ/kg DM)	18.3	18.1	17.9	17.7
**Fatty acid profile (% total FA)**				
14:0	0.38	0.40	0.39	0.41
16:0	12.6	13.2	13.8	14.8
16:1c9	0.20	0.23	0.27	0.33
18:0	3.14	2.96	2.80	2.65
18:1c9	24.8	23.9	23.1	22.5
18:1c11	0.93	1.15	1.36	1.59
18:2n-6	54.5	52.9	50.9	48.4
18:3n-6	n.d.	0.16	0.32	0.49
18:3n-3	1.79	2.99	4.18	5.31
18:4n-3	0.04	0.40	0.80	1.19
20:3n-6	n.d.	0.05	0.12	0.19
20:4n-6	n.d.	0.04	0.08	0.12
20:5n-3	n.d.	0.25	0.54	0.81
Total (% DM)	5.45	4.54	3.95	3.62
**Mineral content (% DM)**				
Calcium	1.00	1.05	1.03	1.14
Phosphorus	0.73	0.78	0.79	0.83
Potassium	1.26	1.39	1.45	1.57
Sodium	0.34	0.61	0.84	1.09
Sulphur	0.29	0.34	0.39	0.44
Magnesium	0.18	0.24	0.29	0.37
Cooper	0.02	0.02	0.02	0.01
Iron	0.03	0.04	0.06	0.08
Manganese	0.01	0.01	0.01	0.01
Zinc	0.02	0.02	0.02	0.02

Abbreviations: DM = dry matter; FA = fatty acid; n.d. = not detected. ^1^ Control = diet without *Tetraselmis* sp.; TSM5 = 95% control diet and 5% *Tetraselmis* sp.; TSM10 = 90% control diet and 10% *Tetraselmis* sp.; TSM15 = 85% control diet and 15% *Tetraselmis* sp. ^2^ Lys min. 98%, ^3^ Met min. 99%, ^4^ Thr min. 98.5%, ^5^ Mineral and vitamin mixture supplied per kg of diet: 6500 UI of vitamin A; 1500 UI of vitamin D_3_; 15.0 mg of vitamin E; 1.0 mg of vitamin K_3_; 1.0 mg of vitamin B_1_; 3.0 mg of vitamin B_2_; 6.0 mg of vitamin B_6_; 0.02 mg of vitamin B_12_; 10.0 mg of pantothenic acid; 15.0 mg of nicotinic acid; 0.5 mg of folic acid; 0.03 mg of biotin; 115.0 mg of betaine; 20.0 mg of vitamin C; 100.0 mg of copper; 100 mg of iron; 0.5 mg of iodine; 50.0 mg of manganese; 0.15 mg of selenium; 100 mg of zinc; 3 mg of butylated hydroxytoluene. ^6^ Calculated considering the 4.78 conversion factor for the contribution of the microalgae N [18] and 6.25 for all the other ingredients.

**Table 2:** *Tetraselmis* sp. composition, gross energy content, fatty acid profile, and mineral content.

	*Tetraselmis* sp.
**Chemical composition (% DM)**	
Dry matter (%)	97.1
Ash	33.4
Organic matter	66.6
N	5.8
Crude protein (N × 6.25)	36.2
Crude protein (N × 4.78) ^1^	27.7
Ether extract	7.4
Neutral detergent fiber	10.7
Gross energy (MJ/kg DM)	14.8
**Fatty acid profile (% total FA)**	
14:0	0.71
16:0	28.7
16:1c9	1.29
18:0	0.36
18:1c9	8.54
18:1c11	5.94
18:2n-6	4.79
18:3n-6	3.73
18:3n-3	27.4
18:4n-3	9.04
20:3n-6	1.70
20:4n-6	0.83
20:5n-3	6.22
Total (% DM)	1.66
**Mineral content (% DM)**	
Calcium	1.40
Phosphorus	1.20
Potassium	3.12
Sodium	3.97
Sulphur	1.24
Magnesium	1.24
Cooper	0.001
Iron	0.32
Manganese	0.005
Zinc	0.01

Abbreviations: DM = dry matter; N = nitrogen; FA = fatty acid. ^1^ Conversion factor for microalgae [18].

**Table 3:** Effect of dietary *Tetraselmis* sp. inclusion levels on feed intake, growth parameters of piglets, and fecal dry matter content.

	Diets ^1^				SEM	*p*-Values ^2^	
	Control	TSM5	TSM10	TSM15		L	Q
Initial weight (kg)	11.9	11.5	12.0	11.3	0.67	0.634	0.836
Final weight (kg)	17.9	17.2	17.6	15.4	0.87	0.087	0.416
DM intake (g/d)	562	514	513	448	21.4	0.002	0.711
ADG (g)	426	407	398	295	36.4	0.025	0.270
Fecal DM (g/kg)	324	317	276	318	16.7	0.426	0.172

Abbreviations: SEM = standard error of the mean; ADG = average daily gain; DM = dry matter. ^1^ Control = diet without *Tetraselmis* sp.; TSM5 = 95% control diet and 5% *Tetraselmis* sp.; TSM10 = 90% control diet and 10% *Tetraselmis* sp.; TSM15 = 85% control diet and 15% *Tetraselmis* sp. ^2^ *p*-values for L = linear polynomial contrast and Q = quadratic polynomial contrast.

**Table 4:** Effect of dietary inclusion levels of *Tetraselmis* sp. on the diets’ apparent total tract digestibility.

	Diets ^1^				SEM	*p*-Values ^2^	
	Control	TSM5	TSM10	TSM15		L	Q
**ATTD (%)**							
Dry matter	88.6	87.9	87.0	85.5	0.45	<0.001	0.322
Ash	67.2	70.8	72.6	75.7	0.98	<0.001	0.833
Organic matter	89.9	89.2	88.3	86.6	0.45	<0.001	0.186
N	85.3	84.2	83.7	82.3	0.60	0.002	0.830
Ether extract	83.7	84.8	81.8	77.6	1.06	<0.001	0.022
Neutral detergent fiber	62.2	68.3	71.7	69.9	1.46	<0.001	0.014
Acid detergent fiber	44.2	45.7	42.7	45.2	2.97	0.994	0.886
Gross energy	88.1	87.3	86.0	84.1	0.50	<0.001	0.283
Fatty acids							
16:0	87.3	81.8	73.1	67.8	1.43	<0.001	0.930
16:1c9	96.6	95.7	93.4	91.6	0.74	<0.001	0.504
18:0	19.1	−15.0	−59.5	−77.9	11.2	<0.001	0.500
18:1c9	98.5	97.6	96.1	94.1	0.66	<0.001	0.404
18:1c11	96.0	94.3	90.6	88.6	1.05	<0.001	0.875
18:2n-6	99.4	99.0	98.4	97.3	0.41	0.002	0.383
18:3n-3	99.2	99.0	98.8	98.0	0.27	0.001	0.269
20:5n-3	-	98.5	99.1	98.7	0.32	0.696	0.280
Total FAs	92.9	89.8	85.5	83.2	0.76	<0.001	0.641
Minerals							
Calcium	64.7	65.5	67.7	63.2	2.31	0.822	0.260
Phosphorus	66.7	67.4	65.8	58.9	2.97	0.083	0.221
Potassium	78.7	79.6	81.5	80.1	1.53	0.390	0.430
Sodium	81.1	82.8	83.7	83.9	2.06	0.328	0.724
Sulphur	75.5	73.4	71.0	64.8	2.34	0.005	0.387
Magnesium	32.6	38.9	39.6	33.4	5.44	0.895	0.263
Cooper	23.3	33.4	38.8	23.5	6.63	0.843	0.071
Iron	47.5	45.3	49.6	44.4	4.88	0.274	0.200
Manganese	28.9	14.6	20.4	14.4	7.77	0.299	0.601
Zinc	−40.8	−50.3	−29.6	−43.7	9.66	0.787	0.818

Abbreviations: SEM = standard error of the mean; ATTD = apparent total tract digestibility; FA = fatty acid. ^1^ Control = diet without *Tetraselmis* sp.; TSM5 = 95% control diet and 5% *Tetraselmis* sp.; TSM10 = 90% control diet and 10% *Tetraselmis* sp.; TSM15 = 85% control diet and 15% *Tetraselmis* sp. ^2^ *p*-values for L = linear polynomial contrast and Q = quadratic polynomial contrast.

**Table 5:** Effect of dietary inclusion levels of *Tetraselmis* sp. on the nitrogen balance of piglets and the diets’ digestible and metabolizable energy contents.

	Diets ^1^				SEM	*p*-Values ^2^	
	Control	TSM5	TSM10	TSM15		L	Q
Ingested N (g/d)	16.8	15.9	15.3	14.0	0.98	0.058	0.829
Fecal excreted N (g/d)	2.5	2.6	2.5	2.5	0.21	0.886	0.760
Urinary excreted N (g/d)	1.9	1.8	2.7	2.5	0.16	0.001	0.832
Retained N (g/d)	12.5	11.5	10.2	9.0	0.72	0.002	0.874
N retention efficiencies (%)							
NRC ^3^	86.9	86.5	77.9	76.6	1.71	<0.001	0.788
NPRC ^4^	74.1	72.5	65.4	63.0	1.82	<0.001	0.824
Energy values of diets							
DE (MJ/kg DM)	13.0	12.9	12.7	12.4	0.74	<0.001	0.283
ME (MJ/kg DM)	12.7	12.6	12.4	12.1	0.72	<0.001	0.283
ME/DE	0.983	0.985	0.975	0.968	0.002	<0.001	0.104

Abbreviations: DE = digestible energy; ME = metabolizable energy. ^1^ Control = diet without *Tetraselmis* sp.; TSM5 = 95% control diet and 5% *Tetraselmis* sp.; TSM10 = 90% control diet and 10% *Tetraselmis* sp.; TSM15 = 85% control diet and 15% *Tetraselmis* sp. ^2^ *p*-values for L = linear polynomial contrast and Q = quadratic polynomial contrast.^3^ NRC = nitrogen retention coefficient = (retained N/absorbed N) × 1000. ^4^ NPRC = nitrogen practical retention coefficient = (retained N/ingested N) × 1000.

**Table 6:** Estimated values of apparent total tract digestibility and energy content of the *Tetraselmis* sp. biomass used in experimental diets.

	Estimated Values ± SEs	Confidence Limits
**ATTD (%)**		
Dry matter ^1^	68.3 ± 3.86	60.3 ↔ 76.3
Organic matter ^1^	68.4 ± 3.57	61.0 ↔ 75.8
N ^2^	66.1 ± 5.11	55.5 ↔ 76.7
Ether extract ^1^	41.1 ± 9.56	21.3 ↔ 61.0
Gross energy (GE) ^1^	61.3 ± 4.28	52.4 ↔ 70.2
Fatty acids		
16:0 ^1^	−45.4 ± 11.69	−70.2 ↔ −20.6
16:1c9 ^1^	62.0 ± 5.84	49.6 ↔ 74.3
18:0 ^1^	−638 ± 100.2	−850 ↔ −426
18:1c9 ^1^	69.2 ± 4.93	58.7 ↔ 79.6
18:1c11 ^1^	44.9 ± 8.21	27.5 ↔ 62.3
18:2n-6 ^1^	85.1 ± 3.19	78.3 ↔ 91.8
18:3n-3 ^1^	91.4 ± 2.11	86.9 ↔ 95.9
20:5n-3 ^2^	98.8 ± 0.21	98.4 ↔ 99.1
Total FAs ^1^	26.4 ± 6.31	13.0 ↔ 30.8
**Energy content**		
DE (MJ/kg DM) ^1^	9.0 ± 0.63	7.8 ↔ 10.4
ME (MJ/kg DM) ^1^	8.8 ± 0.62	7.6 ↔ 10.1
ME/DE ^1^	0.876 ± 0.0226	0.829 ↔ 0.923
**DCP (% DM) ^3^**	18.3	-

Abbreviations: ATTD = apparent total tract digestibility; DM = dry matter; DE = digestible energy; ME = metabolizable energy; DCP = digestible crude protein. ^1^ Estimates of the standard errors and confident limits obtained from regression equations of the incorporation of *Tetraselmis* sp. on the ATTD solved to 100%. ^2^ When no linear effect of the dietary incorporation of *Tetraselmis* sp. was observed, the estimates of the standard errors and confident limits were obtained from the common LSMeans estimate of the ATTD values of the treatments containing *Tetraselmis* sp. ^3^ DCP = (N × N_ATTD) × 4.78.

**Table 7:** Effect of dietary inclusion levels of *Tetraselmis* sp. on the viscosity and pH of gastrointestinal contents and small intestine histomorphology of piglets.

	Diets ^1^				SEM	*p*-Values ^2^	
	Control	TSM5	TSM10	TSM15		L	Q
**Viscosity (** **mPa·s)**							
Duodenum + Jejunum	3.67	3.12	3.57	3.28	0.110	0.464	0.565
Ileum	4.68	4.53	4.57	4.83	0.100	0.624	0.352
**pH**							
Stomach	5.39	5.19	5.02	4.78	0.148	0.158	0.943
Duodenum + Jejunum	5.45	5.26	5.71	5.80	0.066	0.002	0.156
Ileum	6.70	6.72	6.66	6.91	0.044	0.129	0.174
Caecum	5.95	5.71	5.80	5.70	0.069	0.324	0.649
Colon	6.16	6.22	6.17	6.01	0.055	0.347	0.356
**Intestinal histomorphology**							
**Height of the villi (** **μm** **)**							
Duodenum	340	398	393	361	22.1	0.782	0.345
Jejunum	404	413	392	381	18.1	0.609	0.794
Ileum	280	330	363	414	14.5	<0.001	0.983
**Width of the villi (μm)**							
Duodenum	159	162	181	174	4.6	0.110	0.585
Jejunum	135	144	153	150	4.9	0.259	0.600
Ileum	175	167	171	157	5.9	0.361	0.818
**Depth of the crypts (μm)**							
Duodenum	429	453	397	445	15.2	0.955	0.718
Jejunum	310	346	310	309	12.6	0.742	0.482
Ileum	259	254	277	295	9.6	0.143	0.549
**Villi/crypts ^3^**							
Duodenum	0.80	0.91	0.99	0.80	0.05	0.833	0.171
Jejunum	1.33	1.22	1.25	1.32	0.07	0.984	0.556
Ileum	1.11	1.32	1.32	1.40	0.05	0.032	0.460

Abbreviations: SEM = standard error of the mean. ^1^ Control = diet without *Tetraselmis* sp.; TSM5 = 95% control diet and 5% *Tetraselmis* sp.; TSM10 = 90% control diet and 10% *Tetraselmis* sp.; TSM15 = 85% control diet and 15% *Tetraselmis* sp. ^2^ *p*-values for L = linear polynomial contrast and Q = quadratic polynomial contrast. ^3^ Height of villi/depth of crypts.

**Table 8:** Effect of dietary *Tetraselmis* sp. inclusion levels in piglets’ caecum and colon volatile fatty acid concentrations and profiles.

	Diets ^1^				SEM	*p*-Values ^2^	
	Control	TSM5	TSM10	TSM15		L	Q
**Caecum**							
**Total VFAs (mmol/L)**	24.5	20.9	20.7	20.4	2.33	0.243	0.494
**VFA profile (mmol%)**							
C2:0	54.9	52.0	55.3	59.1	1.82	0.066	0.083
C3:0	24.9	28.2	30.0	26.0	2.16	0.603	0.107
C4:0	16.8	16.2	10.8	11.1	1.84	0.015	0.821
C5:0	2.57	2.06	1.62	1.07	0.50	0.042	0.966
C6:0	0.29	0.15	0.16	0.37	0.07	0.400	0.017
iso-C4:0	0.47	0.93	1.59	1.60	0.21	0.005	0.375
iso-C5:0	0.32	0.51	0.53	0.90	0.13	0.009	0.512
**Colon**							
**Total VFAs (mmol/L)**	2.28	3.14	3.22	5.46	0.85	0.023	0.431
**VFA profile (mmol%)**							
C2:0	70.0	77.1	68.2	66.7	4.33	0.340	0.334
C3:0	13.2	13.9	17.1	19.7	2.79	0.092	0.742
C4:0	11.8	8.49	11.1	9.87	1.89	0.706	0.580
C5:0	2.55	1.34	1.88	1.42	0.50	0.209	0.473
C6:0	1.66	1.20	1.16	0.65	0.22	0.006	0.911
iso-C4:0	n.d.	n.d.	n.d.	n.d.	-	-	-
iso-C5:0	1.19	0.98	1.24	1.13	0.30	0.964	0.876

Abbreviations: SEM = standard error of the mean; VFA = volatile fatty acid; n.d. = not detected. ^1^ Control = diet without *Tetraselmis* sp.; TSM5 = 95% control diet and 5% *Tetraselmis* sp.; TSM10 = 90% control diet and 10% *Tetraselmis* sp.; TSM15 = 85% control diet and 15% *Tetraselmis* sp. ^2^ *p*-values for L = linear polynomial contrast and Q = quadratic polynomial contrast.

## Data Availability

The original contributions presented in this study are included in this article. Further inquiries can be directed to the corresponding author/s.
